# Knowledge gaps in extracorporeal blood purification: what would be required for its successful application in septic shock?

**DOI:** 10.1186/s40635-025-00819-8

**Published:** 2025-11-21

**Authors:** Sascha David, Thomas Rimmelé, Michael Joannidis, Massimo Girardis, Peter Pickkers, Nathan D. Nielsen, Alix Buhlmann, Zsolt Molnar, Marlies Ostermann, Jan T. Kielstein, Pedro David Wendel-Garcia, Christian Bode, Klaus Stahl

**Affiliations:** 1 Department of Nephrology & Hypertension, Medical School Hannover, Hannover, Germany; 2 Institute of Intensive Care Medicine, University Hospital Zurich and University of Zurich, Rämistrasse 100, CH-8032 Zurich, Switzerland; 3 Department of Anaesthesiology and Critical Care Medicine, Nice University Hospital, Nice, France; 4 Division of Intensive Care and Emergency Medicine, Department of Internal Medicine, Medical University of Innsbruck, Innsbruck, Austria; 5 Anaesthesiology and Intensive Care Department, University Hospital of Modena, University of Modena, Reggio Emilia, Italy; 6 Department Intensive Care, Radboud University Medical Center, Nijmegen, The Netherlands; 7 Division of Pulmonary, Critical Care and Sleep Medicine, Department of Medicine, University of New Mexico School of Medicine, Albuquerque, USA; 8 Section of Transfusion Medicine and Therapeutic Pathology, Department of Pathology, University of New Mexico School of Medicine, Albuquerque, USA; 9 Department of Anesthesiology and Intensive Therapy, Semmelweis University, Budapest, Hungary; 10 Department of Anesthesiology and Intensive Therapy, Poznen University of Medical Sciences, Poznen, Poland; 11 Department of Critical Care, Guy’s and St Thomas’ NHS Foundation Trust, King’s College London, London, UK; 12 Medical Clinic V: Nephrology and Blood Purification, Hospital Brunswick, Brunswick, Germany; 13 Division of Cardiothoracic Anesthesia and Intensive Care Medicine, Department of Anesthesiology, General Intensive Care and Pain Medicine, Medical University of Vienna, Vienna, Austria; 14 Department of Anaesthesiology and Intensive Care Medicine, University Hospital Bonn, Bonn, Germany; 15 Department of Gastroenterology, Hepatology, Infectious Diseases and Endocrinology, Hannover Medical School, Hannover, Germany

**Keywords:** Adsorption, Cytokines, Hemoadsorption, Host response, Plasma exchange, Polymyxin, Shock

## Abstract

Sepsis remains a leading cause of death worldwide, characterized by a dysregulated host response to infection that results in organ dysfunction. Extracorporeal blood purification (EBP) therapies traditionally aim to remove circulating mediators involved in this pathological response, although novel technologies that can remove cells and even living pathogens have recently been developed. Despite their growing clinical use, robust evidence supporting EBP in septic shock as an adjuvant therapy is lacking, and several knowledge gaps hinder their effective and safe application. This narrative review critically examines these gaps from both mechanistic and clinical perspectives. Key issues include the dynamic and compartmentalized nature of the immune response, the unclear roles of specific cytokines, and the potential removal of protective anti-inflammatory mediators. Broad-spectrum adsorption may induce unintended immunomodulatory effects, including desorption and altered leukocyte trafficking. Selective approaches, such as endotoxin removal with polymyxin B hemoadsorption, face challenges related to dose, patient stratification, and the limitations of endotoxin activity assays. Therapeutic plasma exchange offers the potential to restore homeostasis but raises questions regarding optimal regimens, replacement fluids, and the risk of unintended drug clearance. The heterogeneity of trial designs, insufficient patient phenotyping, and variability in treatment protocols have led to inconclusive or conflicting clinical outcomes, including some trials suggesting potential harm. This review underscores the need for better mechanistic understanding, real-time immune monitoring, and ideally targeted clinical trial designs to define which patients might benefit from EBP and when. Ultimately, the path to effective application of EBP in sepsis lies in individualized therapy guided by immune profiling, biomarker-driven stratification, and rigorous evaluation in high-quality randomized controlled trials.

## Introduction

Mortality in patients with sepsis remains exceedingly high, with one out of every five deaths worldwide attributed to it [1]. Sepsis is not a distinct disease but rather a complex syndrome that is characterized by a pathological host response to an infection leading to life-threatening organ dysfunctions [2]. Despite widespread recognition of the central role of such a dysregulated host response no specific targeted therapeutic strategy, such as the blockade of single mediator pathways, has been proven effective [3]. An alternative approach, targeting the removal of either multiple mediators (e.g., cytokines) at once or upstream triggers, e.g., damage (DAMPs) or pathogen (PAMPs) associated molecular patterns*,* could be more effective.

In the most severely critically ill patients who fail to improve after standard treatment, intensivists are often tempted to consider rescue approaches in the form of adjuvant therapies, despite their experimental status [4, 5]. The term *extracorporeal blood purification* (EBP) encompasses techniques that aim to remove and/or modulate circulating substances to achieve physiological homeostasis, including support of the function of specific organs and/or detoxification [6, 7].

Specific acute EBP techniques include renal replacement therapy, isolated ultrafiltration, hemoadsorption, and plasma therapies, all of which can be applied as stand-alone therapy or in combination. Adsorption can be done from whole blood, termed hemoadsorption (HA, Table 1) or from separated plasma, mostly done as coupled plasma filtration and adsorption (CPFA). HA can selectively target single solutes (e.g., endotoxin), or non-selectively target molecules with certain biochemical and physical properties (e.g., cytokines, chemokines, and DAMPs). Some products containing specific heparin-treated polymer beads can even remove pathogens (e.g., bacteria and viruses) [8].

**Table 1:** Common abbreviations in the field of adjuvant blood purification

Abbreviation	Full text	Explanation
EBP	Extracorporeal blood purification	General term for techniques that use an extracorporeal circuit to remove circulating substances
HA	Hemoadsorption	Technology that adsorbs certain solutes dependent on their size, charge and hydrophobicity
Broad-spectrum HA	Broad-spectrum hemoadsorption	Adsorption of a broad group of solutes only selective regarding their physical properties (above)
Selective HA	Selective hemoadsorption	Adsorption of a specific molecule, e.g., endotoxin
TPE	Therapeutic plasma exchange	Apheresis of plasma for broad-spectrum removal of solutes and replacement of consumed plasma proteins
CPFA	Coupled plasma filtration and adsorption	Apheresis of plasma followed by broad-spectrum adsorption of the patients` plasma

**Fig. 1:**
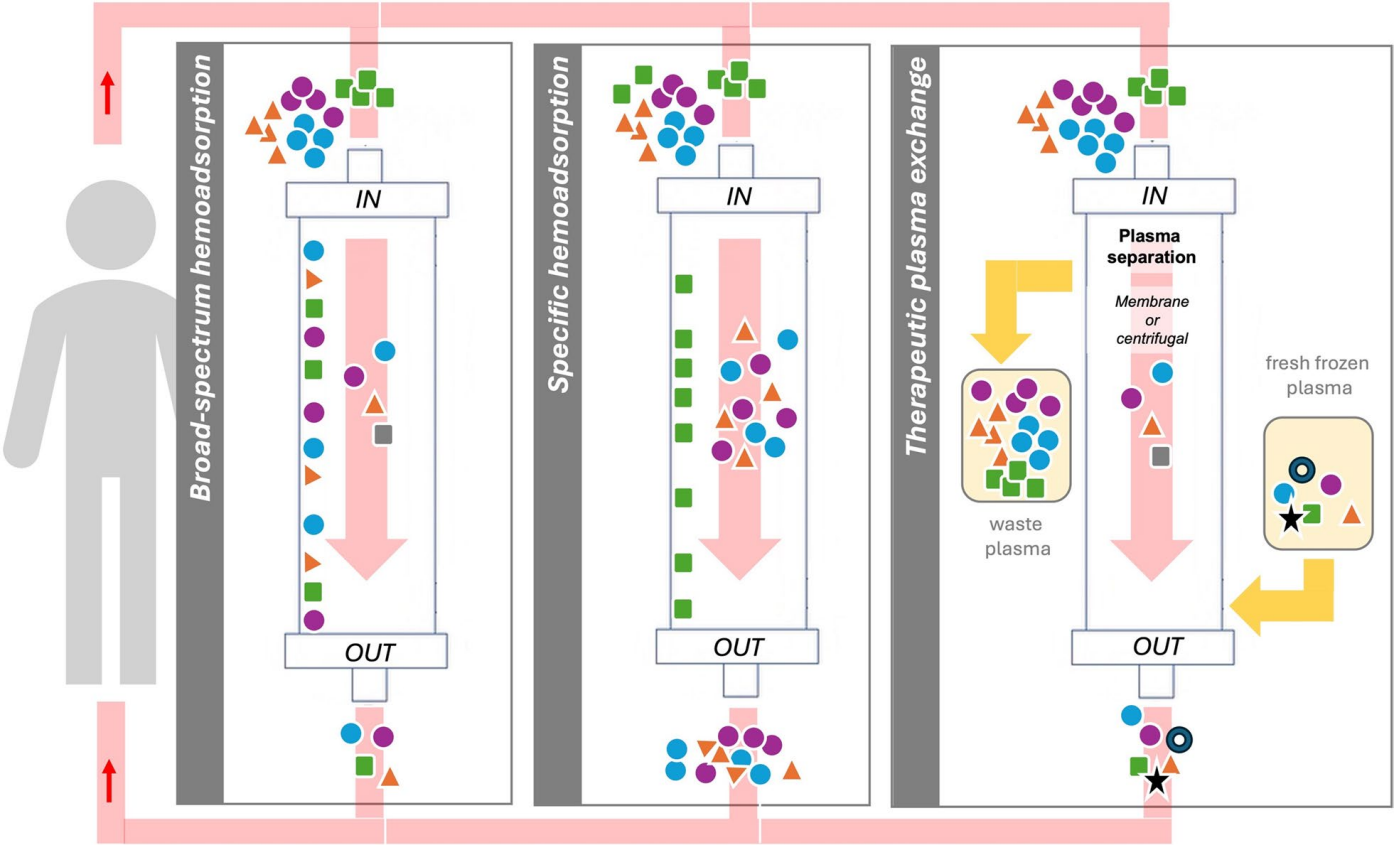
Schematic overview highlighting the principles of the three extracorporeal blood purification strategies reviewed. Broad-spectrum hemoadsorption (left): multiple substances (colored squares, circles and triangles) are adsorbed in the cartridge; specific hemoadsorption (middle): only one substance schematized as green squares (e.g., representative for endotoxin) is adsorbed in the cartridge; plasma exchange (right): multiple substances (colored squares, circles and triangles) are removed within the waste plasma, whereas the normal repertoire of plasma proteins will be supplemented by the fresh frozen plasma from healthy volunteers. This includes molecules that are lacking due to disease consumption (schematized as a black star, representative, e.g., ADAMTS13)

A recent survey among approximately 100 European critical care physicians demonstrated that almost 75% of participants are using adjuvant EBP despite the lack of a clear recommendation for its use [9]. Several different HA devices have been studied in preclinical models and many of them are already in clinical use (Fig. 1) [10].

Here, we aim to review theoretical ideas and recent findings—both mechanistic and clinical—about the knowledge gaps in EBP in sepsis that need to be addressed to make it potentially effective at the bedside. We will focus on 1) general knowledge gaps, 2) broad-spectrum HA, 3) selective HA and 4) therapeutic plasma exchange (TPE).

## Part 1: General knowledge gaps related to blood purification strategies

### Taming the fire: *should we really interfere with inflammation?*

Simplified, the response of the immune system to any infection consists of two key components: pro-inflammation and compensatory anti-inflammation (or immunosuppression), which are likely to occur simultaneously to effectively combat an infection and to balance the organism’s response with the aim to restore homeostasis [11]. However, it is unclear how this interaction becomes maladaptive and thus injurious. This complexity is heightened further by its dynamic and non-linear nature and our limited ability to adequately monitor an individual patient’s immune status. There is also a lack of knowledge about the individual role of certain cytokines as well as their network interaction during the so-called dysregulated host response [12]. Numerous cytokines might be upregulated as part of an adaptive, i.e., required, non-injurious immune response to an infection. The million-dollar question is how to identify the tipping point at which inflammation shifts from being adaptive and beneficial to becoming excessive and harmful or maladaptive [13]. Although excessive inflammation and associated collateral tissue damage are associated with impaired clinical outcomes in sepsis, it seems fair to ask whether interfering with such an evolutionarily conserved biological process is a safe thing to do.

It is likely that people could not survive infections without the ability to produce an inflammatory response [14]. The knowledge of what characterizes an excessive nature of inflammation is of utmost importance to the application of EBP to avoid interference with appropriate inflammatory factors. While removing inflammatory mediators from the patient’s circulation, we risk overlooking the possibility that a localized inflammatory response, which has suddenly sequestered into the bloodstream by generating large gradients, may be crucial required for effective local infection control [15]. On the other hand, it has been demonstrated in an experimental sepsis rat model that modulation of chemokine gradients can also beneficially modulate leukocyte trafficking into the lung [16].

### Are cytokines the critical component of the so-called “pathological host response” that should be focused on?

Interleukin-6 (IL-6) is perhaps the most frequently analyzed cytokine, likely due to its longer half-life and the broad availability of measurement assays making it sort of a “reference cytokine” in the field. Many uncontrolled trials in the field of EBP have shown significant reductions in IL-6 levels following EBP treatments (e.g., [17, 18]), although clearance rates are less frequently reported [19]. The ability of various EBP technologies to eliminate cytokines from the circulation was demonstrated years ago in brain-dead organ donors [19]. However, whether these interventions significantly accelerate the reduction of systemic cytokine levels beyond what is expected from natural disease kinetics remains unclear. In a human endotoxemia model, cytokine clearance rates using HA have been reported to range between 20 and 30 mL/min, accompanied by a blunted cytokine peak response [20]. While these findings are intriguing, the endotoxemia model does not fully replicate clinical sepsis, particularly in key aspects relevant to EBP. Most notably, this model lacks a localized infection as a starting point and instead involves only bloodstream endotoxemia. Nonetheless, preclinical studies suggest that cytokine elimination in sepsis models is feasible [21, 22]. However, rodent sepsis models have significant limitations, and to date, randomized controlled trials (RCTs) in actual septic patients have not demonstrated a consistent reduction in circulating cytokine levels attributable to EBP [23, 24]. Given the rapid endogenous cytokine clearance in case of adequate source control [25], any additional effect of EBP on circulating cytokine levels can be hard to determine.

*Considerations for future trials*: Many trials have been criticized for their lack of patient stratification for predictive enrichment. Since many biomarkers in septic patients often decline during standard treatment alone due to the natural time course of immune response, including such “responders” in an adjuvant rescue trial would add little value. Therefore, future trials could focus on patients with worsening biomarker kinetics instead. In addition, DAMPs that are downstream mediators compared to cytokines and PAMPs can also trigger and maintain sepsis-induced tissue injury and stress and might, therefore, be a potential target for improving the efficacy of immune modulation by EBP in sepsis [18, 26].

### Anti-inflammatory response—*should* we interfere with protective compensatory mechanisms?

Any broad spectrum, or non-selective EBP technology will also interfere with the anti-inflammatory, i.e., compensatory, response. Among others, IL-10, CD64 or monocytic HLA–DR expression has recently gained attention [27–29]. These anti-inflammatory cytokines are usually part of the physiological shift towards immune restauration, adaptation and hemostasis [30] but can also be removed by EBP. For example, it has been reported that broad-spectrum HA as well as TPE removes anti-inflammatory cytokines, such as IL-1ra, IL-4, IL-10 and IL-13 [21, 31, 32], thus potentially negating the effect of removing pro-inflammatory mediators and contributing to reduced effectiveness. On the other hand, some data suggest that the principle of EBP is concentration-dependent clearance. As the concentration of a mediator falls, removal is less efficient. As a result, EBP (unlike a drug) could be seen as self-regulating [33]. This theory, however, neglects the facts that a) high circulating cytokine levels can lead to receptor downregulation, and b) cytokines have distinct potencies (e.g., TNFα and interferon are very potent, whereas IL-10 is relatively weak).

Nevertheless, the theoretical rationale to apply EPB is based on the idea that, during the initial phase of a dysregulated immune response, the production of pro-inflammatory cytokines overwhelms. In this context, bulk removal of molecules is expected to eliminate more pro- than anti-inflammatory cytokines, thereby helping to restore balance between the two inflammatory forces. [34]. Altogether, dynamic monitoring of the immune status would be necessary to individualize EBP.

### Compartmentalized immune response—*is blood the right playing field to purify?*

Local inflammatory milieus might significantly differ from the situation in the bloodstream [15, 35, 36]. Yet, we measure inflammation most of the time exclusively in the blood and can only directly access this compartment by EBP. One hypothesis is that EBP might affect local inflammatory mediators in the infected tissue only via the generation of a concentration gradient, i.e., continuous redistribution of inflammatory mediators from the inflamed tissue to the purified blood compartment [37]. As an experimental example, it has been demonstrated that modulation of chemokine gradients by apheresis can redirect leukocyte trafficking [16]. Again, the question remains whether avoidance of leukocyte tissue infiltration is necessarily a good thing, or whether it could have adverse effects for local control of an infection.

### Treatment intensity and timing

The recommended treatment regimens range widely, and are based on theoretical considerations, feasibility, approval regulations and even on cost but rarely on evidence-based medicine. Table 2 summarizes the heterogeneity of their use in large trials. Specific details regarding individual devices will be covered later in this review in their respective sections.

**Table 2:** Baseline characteristics and dose heterogeneity using different EBP technologies in sepsis trials

Study	*N* (intervention/control)	Device and study design	Hours/device run mean amounts of runs	Primary outcome	Result
Brouwer et al. 2019	116 (67/49)	CytoSorb Single-center, Retrospective IPTW-weighted	24 h Ø 2.34 runs	28-day all-cause mortality	Mortality CytoSorb 47.8% vs. CRRT alone 51% *P* = 0.729
Hawchar et al 2019	20 (10/10)	CytoSorb Single-center, RCT open-label	24 h Ø 1 run	Organ dysfunction (SOFA Score) until 48 h	No statistical difference in SOFA Scores in CytoSorb and control group
Rugg et al 2020	84 (42/42)	CytoSorb Single-center, retrospective propensity-score-matched	24 h 1*	Organ failure and vasopressor requirements	No statistical difference in SOFA Score
Schittek et al 2020	76 (43/33)	CytoSorb, single-center, retrospective control group Prospective intervention group	35.5 h (IQR 17,47) Median 1 (IQR 1, 2)	ICU mortality	No statistical difference in risk adjusted hospital mortality rates
Akil et al 2021	20 (13/7)	CytoSorb, single-center retrospective control group Prospective intervention group	24 h 7 pat.: 3 runs; 3 pat.: 2 runs**	30-day mortality	30-day mortality 0% in CytoSorb 57% in control group
Kogelmann et al 2021	502 (198/304)	CytoSorb, Multicenter study Retrospective	/***Ø 2.7 runs	ICU mortality Hospital mortality	No statistical difference in CytoSorb and control group
Wendel-Garcia et al 2021	96 (48/48)	CytoSorb, single-center propensity-score-matched Retrospective control group Prospective intervention group	24 h Ø 3 runs	Vasopressor requirement and ICU mortality	Higher mortality with CytoSorb CytoSorb 67% Control 42%, *p* = 0,024
Bottari et al 2023	30 (17/13)	CytoSorb Single-center Retrospective control group Prospective intervention group Pediatric cohort	24 h median 3, max. 4	reduction in vasopressors after 96 h	Significant larger vasopressor reduction in CytoSorb; no statistical difference in PELOD-2 score and 28-day mortality
Marino et al 2024	35 (11/24)	CytoSorb Single-center Retrospective	24 h min. 2, max. 16, mean 4,73	vasopressor requirement and organ failure over initial 4 d	Significant lower mortality in CytoSorb (45.4%), compared to control (70.8%) by Kaplan–Meier survival analysis at 270 days (*p* = 0.04)
Giménez-Esparza et al 2019	49 (19/30)	oXiris multicenter randomized, prospective, open clinical trial prematurely closed trial	High dose oXiris (treated plasma ≥ 0.20 L/kg/day) for 3 days	Hospital mortality at 28 days and 90 days	28-day mortality in oXiris 57.9% and control 46.7% (*p* = 0.444); 90-day mortality in oXiris 57.9% and control 63.3% (*p* = 0.878)
Wendel et al 2023	20 (10/10)	oXiris bicentric, multi-arm, randomized, controlled trial	48 h of oXiris, changed after 24 h	Reduction of EAA at 72 h	21% of EAA reduction in oXiris and 12% reduction in SOC (*p* = 0.82)
Eden et al. 2022	15	Seraph 100, prospective, multicenter, non-randomized (single arm)	Single treatment, 4 h	Not provided	Inconclusive
Chitty et al. 2022 [94] PURIFY-OBS-1	106 (53/53)	Seraph 100, retrospective cohort study, COVID 19 Matched control cohort	Information not provided (4 h?)	Not provided	Reduced mortality 32.1% vs. 64.2%; *p* = 0.001
Rouse et al. 2025 [95]	33	Seraph 100, COVID19, single arm	4–5 h	SARS-CoV2-RNA titers, inflammation, endothelial injury	No significant reduction in viral RNA titers in plasma
Lacquaniti et al. 2025 [96]	28 (13/15)	Seraph 100, observational, infective endocarditis/ sepsis	4 h	Pathogen removal	Improved clinical surrogates (vasopressor, AKI), shorter ICU stay
Dellinger et al EUPHRATES 2018	450 (224/226)	Polymyxin B Multicenter, randomized controlled trial	90–120 min hemoadsorption + SOC within 24 h of enrollment	Mortality at 28 days	No reduction in mortality
TIGRIS Expected soon	aprox. 150 (100/50)	Polymyxin B Multicenter, randomized clinical trial	90–120 min hemoperfusion + standard of care within 24 h of enrollment	Mortality at 28 days	Expected
Busund et al 2002	106 (54/52)	Plasma exchange, RCT, single center	Plasma exchange within 6 h, repeated once within 24 h in 27 patients	Mortality at 28 days	Significantly higher survival in plasma exchange
David et al 2021	40 (20/20)	Plasma exchange RCT	Standard of care + one single additional plasma exchange	Erly hemodynamic improvement (6 h)	No change
David, Bode, Stahl		Exchange-2 (ongoing mRCT)	1 to 2 treatments 1.2 × plasma volume	28-day mortality	Ongoing
Zarychanski, Rimmer		Plexsis (ongoing mRCT)	Up to 5 treatments 1.0 × plasma volumen		Ongoing

*RCT* randomized controlled trial, *ICU* intensive care unit, *EAA* endotoxin activity assay; *SOC* standard of care

^*^In this study most patients received 1 CytoSorb run, apart from 3 patients who received 2 runs and 1 patient receiving 6 runs

^**^Amount of runs unclear for 3 patients

^***^No information on hours per CytoSorb run

There are limited data on the ideal timing of EBP initiation [38]. Most trials have attempted to recruit patients as early as possible (to cover the excessive cytokine response of the acute phase of septic shock). One recent report analyzed prospective registry data from 82 septic shock patients treated with endotoxin adsorption and compared early (within 60–180 min) vs. late initiation (within 328–748 min)—the early start was associated with better hemodynamic stability [39].

Nevertheless, patients admitted to the ICU are at variable timepoints of their sepsis course. Admission to the ICU almost never equals the actual onset of sepsis. Cytokine levels in general show a large scatter when measured in any critically ill condition reflecting the patients’ individual response to the injury. Even if there are certain significant differences between the study groups (i.e., survivors vs. non-survivors or patients with infection vs. no-infection), one single measurement of any cytokine at any timepoint is difficult to interpret at the bedside. Therefore, a cytokine kinetics-based approach might take us closer to predictive enrichment to identify subpopulations who may benefit most from EBP.

Future considerations: Improved phenotyping of patients’ host responses using point-of-care biomarkers and their kinetics may help address the challenge that time alone is an arbitrary factor in sepsis. To do this, modern artificial intelligence technologies might help us to investigate these issues using so-called *digital twins* technologies [40, 41].

### Thromboinflammation and endothelial dysfunction—what do we need to investigate besides immune modulation?

The host response is not limited to immune phenomena but also encompasses an endothelial and coagulation response and interactions between these systems [42, 43].

A major clinical consequence of endothelial dysfunction is vasoplegia due to nitric oxide (NO) production and consecutive distributive shock [44]. Another clinically feared occurrence is the vascular barrier breakdown, also known as capillary leakage syndrome. Degradation of the endothelial glycocalyx and disassembly of junctional proteins can lead to the development of gaps between adjacent endothelial cells, resulting in the accumulation of fluids in the interstitial space. This edema formation directly contributes to organ failure. Numerous circulating mediators (including growth factors, such as VEGF, Angiopoietin-2, S1P1 and the Robo/Stat system, as well as glycocalyx degrading enzymes such as heparinase-1) have been implicated in the underlying molecular mechanisms [45–47]. Although these are circulating factors, making them in theory accessible to HA, their size and biochemical characteristics often lie outside the range of adsorption with current devices [48]. Novel strategies to rebalance these systems are highly desirable.

The pathophysiology of sepsis-induced coagulopathy (SIC) and disseminated intravascular coagulation (DIC) involves a dysregulated balance between coagulation and fibrinolysis [49]. This syndrome is triggered by a massive expression of tissue factor (TF) along with platelet activation [49, 50]. Excessive microvascular thrombus formation is further aggravated by the endothelial release of pre-stored *von Willebrand factor* (vWF) and the consumption of its cleaving protease ADAMTS13, as well as the anti-thrombotic protein C [51]. The fibrinolytic system is suppressed due to the up-regulation of plasminogen activator inhibitor (PAI)-1, leading to the delayed breakdown of thrombi. Given that neither excess circulating TF nor vWF multimers can be removed with current adsorptive technologies, targeting the host response beyond the immunologic plane—encompassing endothelial dysfunction and coagulation abnormalities—remains a challenge that EBP researchers should focus on in the future.

## Part 2: Knowledge gaps related to broad-spectrum adsorption

### Persevered bioactivity of surface-immobilized molecules

Developments in both biomedical engineering and the science of cytokine molecular dynamics suggest that immobilization of inflammatory proteins through binding on solid scaffolds may stabilize protein structure and preserve or even amplify protein function [52]. As a thought experiment, let us assume that such phenomena could occur in HA, depending on the exposure position of the bioactive epitope of a given target. In that case, the adsorbent medium itself may become reactive, potentially activating nearby immune cells and simultaneously shielding harmful proteins from the body’s natural clearance mechanisms, such as receptor-mediated uptake, liver metabolism, or glomerular filtration. In other words, it is possible that cytokines bound to an adsorptive surface may remain biologically active, potentially then continuing to trigger the inflammatory cascades via surpassing immune cells. This hypothesis still must be tested.

### Consecutive questions along the ideal and personalized dosing

**Fig. 2:**
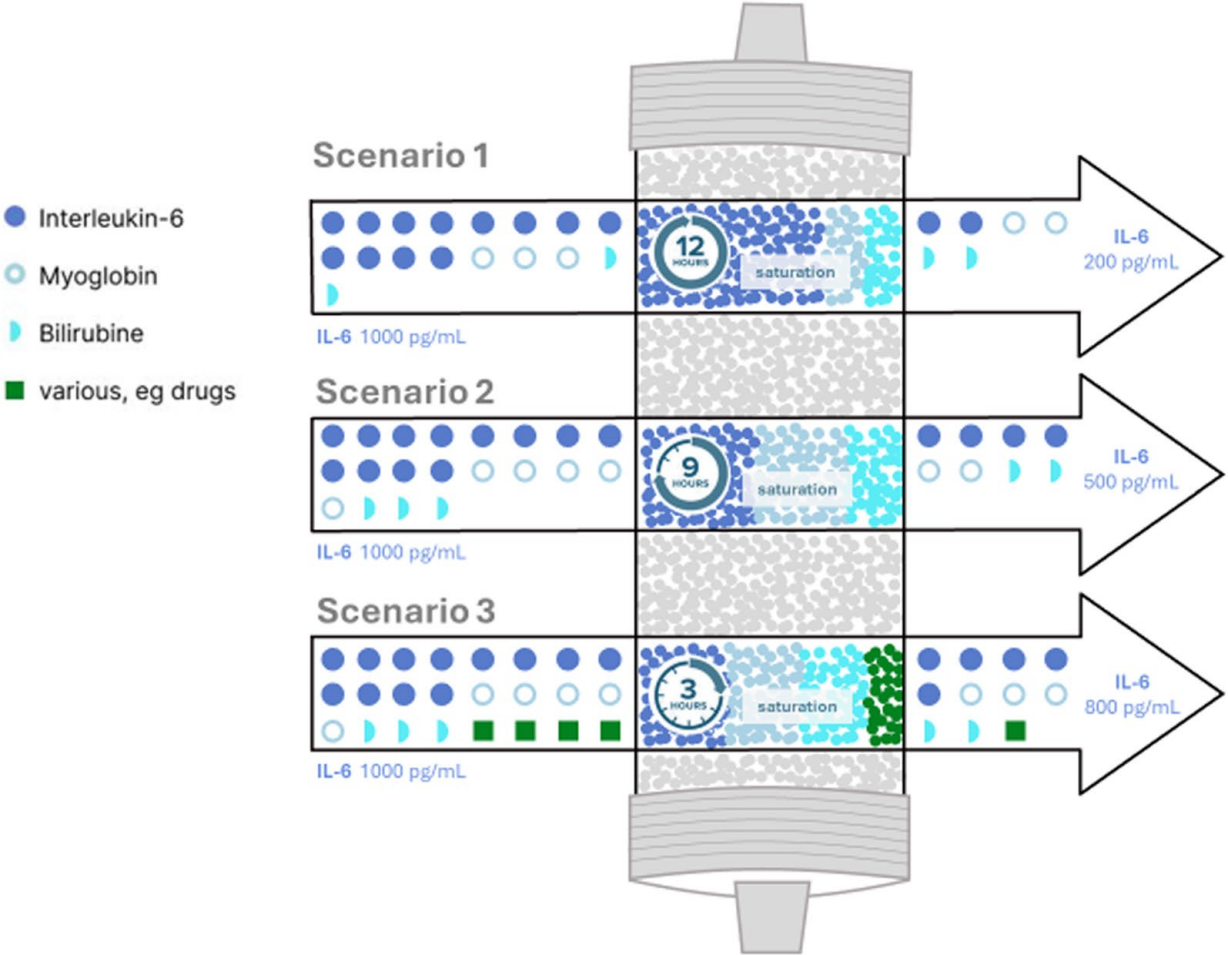
Theoretical saturation kinetics of broad-spectrum adsorption devices. The figure highlights how competitive adsorption from various substances reduces the effective adsorption capacity for a specific one (e.g., IL-6) and shortens the functional lifespan of the cartridge. Three simulated scenarios illustrate the effect of blood composition [as an example interleukin-6 (IL-6), bilirubin, and myoglobin] on the adsorption performance and saturation. All scenarios begin with an initial interleukin-6 (IL-6) concentration of 1000 pg/mL. Scenario 1: The cartridge is exposed primarily to IL-6 (myoglobin, and bilirubin concentrations are low). The device reaches saturation after 12 h and has primarily adsorbed IL-6, effectively reducing IL-6 concentration to 200 pg/mL. Scenario 2: In addition to IL-6, increased levels of myoglobin and bilirubin are present, leading to earlier saturation at 9 h and a less pronounced IL-6 reduction (to 500 pg/mL). Scenario 3: IL-6 is accompanied by bilirubin, myoglobin, and various other compounds (e.g., drugs), causing rapid saturation within 3 h and minimal IL-6 clearance (to 800 pg/mL)

Another determinant is the interval at which adsorptive columns should be replaced due to saturation. From a physics perspective, the adsorptive capacity of a column must eventually be reached [31], depending on the concentration of the target molecule as well as the presence of non-target molecules that can bind competitively. For instance, if the target molecule is IL-6 and serum concentrations are moderately elevated (e.g., 1000 pg/mL), but other solutes (e.g., myoglobin) that can also bind are present at high concentrations, saturation could occur within a matter of hours due to the excess myoglobin leaving the intentionally targeted IL-6 less affected (Fig. 2).

Even more concerning is the observation that adsorbed target molecules may be released back into the patients’ circulation. This phenomenon called desorption could lead to a situation, where systemic concentrations appear lower than the concentrations behind the adsorber (i.e., a negative clearance), effectively resulting in an uncontrolled infusion of potentially harmful molecules back into the patient. Indeed, negative clearance rates of cytokines, such as IL-8, have already been reported. For example, in a human endotoxemia model, desorption of IL-8 over time was documented already after 6 h [20]. Similar findings have been observed in clinical settings for other molecules like myoglobin [53].

*Future considerations*: Work in this area could try to identify models to predict adsorber capacity and time to saturation (e.g., using a handheld mobile app, where a physician can add a patient’s lab values to predict an individualized adsorber exchange interval) either based on real data from clinical trials or on in silico twin designs.

### Problems around heterogeneous results from clinical trials

While numerous case series, small non-randomized studies including registries applying broad-spectrum HA have reported anecdotal improvements of biological and clinical endpoints [17, 54, 55], currently, the best evidence stems from a propensity-score-matched trial that showed a 20% better survival in the intervention group [54]. However, the data are less clear under controlled settings [17, 24, 56–58]. In summary, there is no RCT in sepsis that shows clinical benefits. Neutral trial results may still justify explorative clinical use given all unknowns discussed above that might contribute to the lack of overall success when treating large trial populations rather than treating individual patients. The situation becomes more challenging when high-quality trials have negative (not neutral) results. Along these lines, three well-conducted more recent studies raised serious concerns about the safety of broad-spectrum adsorption in septic patients:

First, the COMPACT-2 RCT testing combined plasma filtration and adsorption (CPFA) also a broad-spectrum adsorptive method—was halted early after 115 patients due to increased mortality (54% vs. 29%) [59]. Second, the ROMPA study (only published in abstract form) aimed to improve on CPFA dose delivery but was also stopped prematurely after enrolling only 49 patients, preventing meaningful analysis [60]. Third, a prospective HA trial with rigorous inclusion criteria (high vasopressor doses, early shock, and high cytokine levels) evaluated the endpoints IL-6 levels, vasopressor need and ICU mortality using a retrospective propensity-score-matched cohort. Surprisingly, the HA group showed a significantly higher mortality (67% vs. 42%) with no improvement in secondary outcomes [23].

These studies suggest that unselective cytokine removal whether through hemo- or plasma adsorption sometimes may have unexplained harmful effects in certain patients. This underlines the need for (a) individualized patient selection and (b) a deeper understanding of the complex biology underlying the dysfunctional host response to target it effectively.

## Part 3: Knowledge gaps related to selective endotoxin adsorption

Different devices have been developed to remove endotoxin from the circulation (e.g., Polymyxin B, Efferon LPS Neo, Altecon LPS etc.). Immobilized Polymyxin B (PMX) has been studied the most. A huge advantage over broad-spectrum removal technologies lies in the knowledge of a single specific target and the possibility to quantify its activity (endotoxin activity (EA)) thus being able to use it as a decision tool to initiate the therapy.

### What endotoxin activity should be targeted by PMX?

Pilot single-center RCTs have shown some clinical benefits [61–63], but the largest multicenter RCT (EUPHRATES) failed to improve survival, although inclusion was stratified based on EA > 0.6 [64]. Although the primary endpoint of 28-day mortality was not significantly different in the entire study population, a secondary analysis investigated subgroups with different EA ranges [65]. The investigators postulated that patients with particularly high EA levels may have exceeded the adsorption capacity In line with this hypothesis, no difference was found in the group with very high EA (> 0.9), but a better MAP, more ventilator-free days and even better survival were observed in the group with moderate EA between 0.6 and 0.89 [65]. The TIGRIS study (NCT03901807) is a follow-up trial investigating if PMX hemoadsorption can improve 28-day mortality in septic shock patients with an EA within exactly that range of ≥ 0.60– < 0.90. The design is based on Bayesian statistics and additionally allows the inclusion of patients from EUPHRATES who fall within this range of EA. The recruitment was completed in Spring 2025, and we are eagerly awaiting the results.

Nevertheless, several important considerations from a clinician’s perspective remain unresolved. A positive trial within a relatively narrow subpopulation cannot be generalized to all patients with septic shock. It is, in fact, the most critically ill patients suffering from septic shock with EA > 0.9 for whom an effective adjuvant (rescue) therapy is most needed. In patients with EA > 0.9 it has been hypothesized that 2 × 2-h treatments might not be sufficient [66].

Nevertheless, the population with EA between 0.6 and 0.89 still experienced high mortality in EUPHRATES (47.2%).

### Is a dose of 2 × 2 h PMX enough for every patient?

The recommended dose for PMX is 2 × 2 h treatments within 24 h. This rather short regimen is based on general adsorption kinetics (i.e., highest effects in the early treatment period due to higher delta between blood and adsorber surface concentrations) as well as the specific endotoxin removal curve of PMX [67, 68]. Adsorption with PMX appears to be so efficient that even high concentrations might be removed within less than 2 h.

However, it is not clear if longer runtimes might be associated with even better adsorption capacities considering potential ongoing endotoxin release from local infectious sites. On the other hand, if one assumes that PMX does saturate within the recommended 2 h, longer runs could also face *desorption* as discussed above for broad-spectrum adsorption. Finally, the manufacturer’s recommendation could also be influenced by practical aspects, such as approval and certification processes.

Some groups from Japan have been able to retrospectively show the safety of up to 12 h of PMX hemoadsorption [69, 70]. These studies seem to indicate a faster hemodynamic stabilization with longer PMX therapy and a trend towards improved survival. Finally, a recent prospective pilot study using a historical RCT as a comparator suggests that extending PMX treatment to 4 h compared to 2 h leads to a greater reduction in EA and IL-6 levels, and is associated with improved hemodynamic recovery [own data, under review]. These results are in line with post-hoc analyses of the EUPHRATES trial which indicate an improved survival in patients with faster EA clearance [71]. Overall, prolonging PMX therapy intervals might be a promising research approach.

### Questions around the endotoxin activity assay (EAA)

While endotoxin levels can be measured in blood, direct measurement by means of the limulus amebocyte lysate (LAL) assay is complex and currently not feasible at the bedside, restricting its broad applicability [72]. Moreover, this assay is only capable of measuring unbound LPS, which, however, is mostly bound in whole blood. The EAA, on the other hand, is a test that draws from the oxidative burst reaction of activated neutrophils when exposed to endotoxin–IgM immune complexes and semi-quantitatively estimates the endotoxin levels via chemoluminescence [73]. The EAA is bedside applicable, but comes with several limitations [68]:

- Short stability of whole blood for testing (< 4 h)
- Linear approximation of EA only between 0.6 and 0.9, after which the relationship becomes exponential
- Limited accuracy if a patient has neutropenia or neutrophil dysfunction

These limitations could explain why trials that employed EAA as an inclusion criterion have been unable to show a significant effect of PMX [69, 70]. Some of the initial trials that only enriched for abdominal sepsis had a positive result [62, 63]. Furthermore, theoretically, activated neutrophils may not immediately reverse their phenotype after endotoxin is removed, which could in part explain the lack of effect of PMX in modulating EAA dynamics over time [74].

Finally, when discussing PMX to remove endotoxin, it should be noted that monoclonal antibodies against endotoxin [75] and modulation of the immune response to endotoxin [76] have been studied since the 1980s. Although, an early single-center RCT [63] as well as a post-hoc analysis in refractory gram-negative septic shock [77] have shown outcome benefits, the larger trials did not confirm this. It could thus certainly be that we do not fully comprehend the immunological effector dynamics of endotoxin and are thus wrongly tackling endotoxin. Nevertheless, it is important to recognize that these studies assumed all patients with GN infections exhibited high levels of circulating endotoxin. We now know this is not the case, suggesting that some of these agents could have been effective if patients had been stratified based on EA.

## Part 4: Knowledge gaps related to plasma exchange

TPE is a more complex method requiring the separation of plasma from the corpuscular blood components followed by the replacement with either human albumin or fresh frozen plasma (FFP). It is a routine procedure to treat diseases like TTP that lack critical plasma components (e.g., the vWF-cleaving protease—ADAMTS13), or diseases that are characterized by pathognomonic circulating factors that need to be removed (e.g., myasthenia gravis and AChR-Ab) [78]. General research priorities for this technology have been reviewed in depth [79]. In the last 20 years, TPE has also been used exploratively in septic shock [80, 81] and two large multicenter RCTs are currently running in Canada (NCT05093075) and Europe (NCT05726825) [82]. The most recent version of the *American Society for Apheresis* (ASFA) guidelines from 2023 categorizes “sepsis with multiorgan failure” as category III (= *optimum role of apheresis therapy is not established*; *decision-making should be individualized.*), and grade 2A (weak recommendation and high quality of evidence) [83].

Besides general questions regarding timing and patient selection, there are also specific knowledge gaps for plasma exchange in septic shock that need to be addressed and might even hinder the potential success of these trials [84]. The clinical evidence has recently been discussed in a pro–con editorial [84, 85].

### What treatment regimens should be implemented?

The fact that the two running RCTs use very different regimens (1–2 vs. up to 5 treatments) underlines the uncertainty in this area. The uncertainty regarding treatment regimens is not unique to sepsis but is a critical question in almost all diseases treated with TPE. Broadly, most TPE centers employ regimens of 3–5 procedures. This number is based on the kinetics of the removal of large (e.g., IgM), and small (e.g., IgG) molecules. For large molecules with a lower volume of distribution (VOD) 1–3 procedures can remove up to 90%. Smaller molecules with a high VOD and significant post-procedure redistribution require 5 or more procedures to reach a similar removal rate [84]. If these considerations are of relevance in sepsis when a given focus of an infection is successfully treated and anti-infectives are given remains unknown. The investigators of EXCHANGE-2 hypothesize that a single (but early) exchange might be sufficient to restore homeostasis and thus interrupt the vicious cycle of organ failure.

### What is the best replacement fluid?

TPE is different from EBP strategies that exclusively remove certain disease mediators [86]. First, the efficacy of removal is less dependent on size, hydrophobicity, or protein binding of a given molecule but mostly on its VOD [87]. Obviously, this broader range of removable targets makes this technology even more susceptible to potentially negative effects by the removal of protective proteins or even drugs. Such a negative effect is theoretically more relevant when septic plasma is replaced with human albumin—TPE using albumin does remove most plasma components, but it can certainly not replace lacking proteins.

If healthy donor plasma (e.g., FFP) is used instead, TPE allows for the correction of acquired deficiencies of protective mediators during the disease process, e.g., anti-permeability [88, 89], anti-thrombotic- [88] and glycocalyx-protective [90] factors as well as deficiencies in immunoglobulins [91]. Thus, the use of plasma as a replacement fluid might have theoretical benefits but it also comes with higher costs, accessibility issues and a higher rate of adverse events (i.e., “transfusion reactions”).

When looking more into the details of plasma products, it becomes obvious that there is also a need to explore differences between plasma products (e.g., single donor FFP and pooled S/D-treated plasma). Maybe even enrichment of plasma for certain coagulation factors (e.g., AT III or ADAMTS13) could be a valuable option. This whole topic has not been looked at in a systematic fashion, but a meta-analysis from 2023 that analyzed TPE using plasma vs. TPE using albumin found a significant mortality benefit only when plasma was used as the replacement fluid [80].

### How should antibiotic doses be adjusted during plasma exchange?

Any broad-spectrum removal has the potential to also undesirably remove drugs. This effect could be very strong for TPE due to its mode of action that is only limited by the VOD and largely independent of the classical drivers of adsorption (size and hydrophobicity) [87]. Proper clinical studies investigating the PK/PD of critical antibiotics peri-TPE are limited to case series [92] and most centers follow theoretical consideration from pharmacologists including additional doses after TPE procedures if the gap between administration and TPE initiation is < 2 h. Therapeutic drug monitoring (TDM) is critically important, and the EXCHANGE-2 trials have a pre-defined sub-study that will quantify antibiotic levels from the waste plasma to get deeper insights into this field [83].

Along these lines, many other beneficial/essential solutes (nutrients, vitamins, etc.) or even drugs other than antibiotics can be removed by TPE and might, therefore, be closely monitored. Improving our understanding of these considerations is of high importance.

### How to interpret circulating mediators of inflammation/infection?

The exchange of diseased plasma from patients with septic shock against healthy plasma can lead to misinterpretation of routine diagnostic biomarkers as these are also removed during TPE. An observational study showed that a single TPE reduced the plasma levels of N-terminal (NT)-pro b-type natriuretic peptide (BNP) (NT-pro BNP) by 23%, C-reactive protein (CRP) by 64%, procalcitonin by 31% and troponin-T by 14% [93].

## Conclusions

EBP is a promising but inadequately understood adjuvant therapeutic approach to modulate the dysregulated host response in septic shock. Table 3 summarizes all discussed knowledge gaps in this review. Despite increasing clinical use, current evidence does not clearly support its routine application, and some trials even suggest potential harm in unselected patients. The complexity of the sepsis pathophysiology demands a more nuanced understanding and patient-specific application of EBP. Broad-spectrum and selective adsorptive therapies, as well as TPE, each face distinct challenges regarding mechanisms, timing, dose, and outcome impact. The potential for unintended removal of protective mediators, desorption, or inadequate targeting further underlines the need for caution. Moving forward, the path to effective and safe use of EBP in sepsis requires rigorous mechanistic research, biomarker-driven patient stratification, and well-designed RCTs. Meaningful integration of EBP into sepsis care is only achievable through individualized, precision-based approaches ideally guided by real-time immune profiling and supported by digital tools such as in silico modeling, *digital twins* and maybe artificial intelligence.

**Table 3:** Knowledge gaps and potential solutions

Knowledge gap	Problem description	Potential solution
1. General considerations		
Interference with inflammation	Unclear when inflammation shifts from adaptive to maladaptive; risk of disrupting protective responses	Develop immune-monitoring tools to define tipping points and guide individualized treatment
Role of cytokines	Uncertain whether cytokine removal meaningfully alters systemic levels or outcomes	Use longitudinal cytokine kinetics and biomarker-based stratification in trials
Impact on anti-inflammatory mediators	Non-selective removal also eliminates potential protective mediators, such as IL-10, thereby negating benefits	Dynamic immune profiling to balance removal of pro- and anti-inflammatory mediators
Compartmentalization of immune response	Local vs. systemic inflammation may differ; EBP acts mainly on blood compartment	Investigate concentration gradients and local tissue effects alongside systemic clearance
Timing and treatment intensity	Optimal initiation time and device runtime are unknown; trials show inconsistent timing strategies	Develop kinetics-based approaches and digital twin models to personalize timing and dosing
Thromboinflammation and endothelial dysfunction	EBP does not address vascular permeability, endothelial dysfunction, and coagulopathy	Expand research beyond cytokines to include endothelial and coagulation pathways
2. Broad-spectrum hemoadsorption		
Preserved bioactivity of bound molecules	Proteins bound to adsorbers might remain biologically active, potentially harmful	Test bioactivity of surface-bound molecules in preclinical/clinical studies
Adsorber dosing and desorption	Unknown saturation kinetics; risk of desorption leading to release of harmful molecules	Develop predictive models and monitoring tools to guide cartridge exchange
Heterogeneous trial results	Conflicting data, including trials showing possible harm with broad-spectrum adsorption	Improve patient stratification and ensure rigorous safety monitoring in RCTs
3. Selective hemoadsorption		
Endotoxin activity threshold	Uncertainty about which EA ranges benefit from PMX treatment	Stratify patients by EA range (e.g., 0.6–0.89) and refine inclusion criteria in trials
PMX dosing duration	Standard 2 × 2 h regimen may be insufficient for high EA; unclear if longer runs are beneficial	Investigate extended treatment durations and optimize based on clearance kinetics
Validity of endotoxin activity assay	EAA has limited accuracy and applicability; may misclassify patients	Improve or replace assays with more reliable bedside diagnostics
3. Therapeutic plasma exchange		
Optimal TPE regimens	Trials use different numbers of exchanges; unclear how many are needed for efficacy	Compare single vs. multiple exchanges in RCTs; adapt based on molecule distribution kinetics
Replacement fluid choice	Albumin vs. plasma leads to different effects; plasma may replenish protective factors but increases risk and cost	Conduct trials comparing replacement fluids; consider enriched plasma products
Drug clearance during TPE	TPE can remove antibiotics and essential drugs, risking underdosing	Perform PK/PD studies; implement therapeutic drug monitoring and dose adjustments
Interpretation of biomarkers	TPE removes biomarkers, such as CRP, PCT, and NT-proBNP, complicating clinical interpretation	Develop adjusted reference ranges or correction factors post-TPE

## Data Availability

Not applicable.

## Abbreviations

ADAMTS13: A disintegrin and metalloproteinase with thrombospondin motifs 13
AKI: Acute kidney injury
ASFA: American society for apheresis
BNP: B-type natriuretic peptide
CPFA: Coupled plasma filtration and adsorption
CRP: C-reactive protein
DAMPs: Damage-associated molecular patterns
DIC: Disseminated intravascular coagulation
EA: Endotoxin activity
EAA: Endotoxin activity assay
EBP: Extracorporeal blood purification
ESICM: European society of intensive care medicine
FFP: Fresh frozen plasma
GN: Gram-negative
HA: Hemoadsorption
ICU: Intensive care unit
IL: Interleukin
LAL: Limulus amebocyte lysate
LPS: Lipopolysaccharide
mRCT: Multicenter randomized controlled trial
NO: Nitric oxide
NT-pro BNP: N-terminal prohormone of B-type natriuretic peptide
PAMPs: Pathogen-associated molecular patterns
PAI-1: Plasminogen activator inhibitor-1
PK/PD: Pharmacokinetics/pharmacodynamics
PMX: Polymyxin B
RCT: Randomized controlled trial
RRT: Renal replacement therapy
S1P1: Sphingosine-1-phosphate receptor 1
SOC: Standard of care
SOFA Score: Sequential organ failure assessment score
SSC: Surviving sepsis campaign
TDM: Therapeutic drug monitoring
TF: Tissue factor
TIGRIS: Targeted intervention with polymyxin B hemoperfusion in sepsis (trial)
TLR-4: Toll-like receptor 4
TPE: Therapeutic plasma exchange
TTP: Thrombotic thrombocytopenic purpura
VOD: Volume of distribution
vWF: Von Willebrand factor
